# Supplementary material for: Glycosylation Pathways Targeted by Deregulated miRNAs in Autism Spectrum Disorder
Source: Int J Mol Sci. 2025 Jan 17;26(2):783. doi: 10.3390/ijms26020783 (PMC11766332; doi:10.3390/ijms26020783)
Supplement: Supplementary file 1 [file ijms-26-00783-s001.zip › ijms-3365929-supplementary.pdf]

Supplementary Material

Supplementary Table S1: Subset of 25 ASD-miRNAs associated with glycosylation pathways and reported to be upregulated or downregulated in patients with ASD in at least two studies.

miRNA	Upregulated (References)	Downregulated (References)	Glycosylation Pathways	Targeted Glycogenes
1. hsa-miR-106b-5p	Abdelkarem et al., 2024; Huang et al., 2021	Safdar et al., 2021	N-Glycan biosynthesis	ALG2, MAN2A1, STT3B, ALG9, MAN1A2, MAN1C1, ALG13
2. hsa-miR-92a	Vaccaro et al., 2018	Huang et al., 2015	Glycan degradation	NEU3
3. hsa-miR-27a-3p	Vasu et al., 2014; Vaccaro et al., 2018; Hicks et al., 2016		Mucin type O-Glycan biosynthesis	GALNT7, B4GALT5, ST3GAL1, GCNT3, GALNT1, GALNT3, GALNT2
4. hsa-miR-21-3p	Wu et al., 2016; Mor et. al 2015; Mor et Shomron, 2013	Abu-Elneel et al., 2008	Glycosaminoglycan biosynthesis - heparan sulfate / heparin (Irie et al., 2012) (Pérez et al., 2016)	EXT1, EXTL3, NDST2
5. hsa-let-7a-5p		Hicks et al., 2020; Mor et Shomron, 2013; Huang et al., 2015; Mor et al., 2015	Glycosaminoglycan biosynthesis - keratan sulfate<br>Other types of O-glycan	B4GALT1, B3GNT1, B4GALT3<br>OGT, POMT2, GXYLT1, LFNG, B4GALT1, EOGT, B4GALT3
6. hsa-miR-199a-5p	Vaccaro et al., 2018; Ghahramani Seno et al., 2011		Glycosaminoglycan biosynthesis - heparan	EXT1

			sulfate / heparin<br>(Irie et al., 2012) (Pérez et al., 2016)	
7. hsa- miR-195-5p	Vasu et al., 2014; Sarachana et al., 2010	Huang et al., 2015	Glycosaminoglycan biosynthesis - chondroitin sulfate / dermatan sulfate<br><br>Mucin type O-Glycan biosynthesis	UST, DSE, CHPF, CHPF2<br><br>GALNT7, GALNT1, GALNT3, GALNT2
8. hsa-miR-132-3p	Talebizadeh et al., 2008	Sarachana et al., 2010; Abu-Elneel et al., 2008	Other types of O-glycan biosynthesis	LFNG, B3GAT1, POMT1, EOGT
9. hsa-miR-132-5p	Talebizadeh et al., 2008; Kim et al., 2023	Sarachana et al., 2010; Abu-Elneel et al., 2008	Glycosaminoglycan biosynthesis - keratan sulfate<br><br>N-Glycan biosynthesis<br><br>Other types of O-glycan biosynthesis	B4GALT1, B3GNT1<br><br>GANAB, B4GALT1<br><br>B4GALT1
10. hsa-miR-155-5p	Almehmadi et al., 2020; Wu et al., 2016; Mor et al., 2013		Other glycan degradation	NEU, GLB1
11. hsa-miR-181a	Hicks et al., 2016; Ghahramani Seno et al., 2011;		Glycosaminoglycan biosynthesis - keratan sulfate; Other types of O-glycan biosynthesis	B4GALT2, B4GALT1, PLOD3, POMGNT1, POMT1

12. hsa-miR-30e-5p		Jyonouchi et al., 2019; Hicks et al., 2016; Sarachana et al., 2010.	Mucin type O-Glycan biosynthesis	GALNT7, B4GALT5, ST3GAL1, GALNT6, GCNT3, GALNT1, GALNT2, GALNT3
13. hsa-miR-30c	Sarachana et al., 2010	Hicks et al., 2016	Mucin type O-Glycan biosynthesis	GALNT7, B4GALT5, ST3GAL1, GCNT3, GALNT1, GALNT3, GALNT2
14. miR-34c-5p	Huang et al., 2015	Bleazard., 2018	Glycosaminoglycan biosynthesis - chondroitin sulfate / dermatan sulfate<br><br>Glycosaminoglycan degradation	CSGALNACT2<br><br>GNS
15. hsa-miR-379-5p	Mor et al., 2015;<br>Hicks et al., 2020		Mucin type O-Glycan biosynthesis	GALNT11
16. hsa-miR-484	Jyonouchi et al., 2017; Wu et al., 2016;	Abu-Elneel et al., 2008	Glycosaminoglycan biosynthesis - chondroitin sulfate / dermatan sulfate	CHST3, CHSY3, XYLT2, CHSY1
17. hsa-miR-15a-5p		Jyonouchi et al., 2017; Kichukova et al., 2017; Huang et al., 2015; Abu-Elneel et al., 2008	Glycosaminoglycan biosynthesis - chondroitin sulfate / dermatan sulfate	UST, B3GALT6, DSE, CHPF, CHPF2
18. hsamiR-15b-5p		Huang et al., 2015; Abu-Elneel et al., 2008	Glycosaminoglycan biosynthesis - chondroitin sulfate / dermatan sulfate	UST, B3GALT6, DSE, CHPF, CHPF2

19. has- miR-1277-3p	Wu et al., 2016; Winden et al., 2018		Glycosaminoglycan biosynthesis - keratan sulfate<br><br>Glycosaminoglycan biosynthesis - heparan sulfate	CHST1, B3GNT2<br><br>EXT1
20. hsa-miR-144-3p	Salloum-Asfar et al., 2021; Nakata et al., 2019; Mor et al., 2015		Other types of O-glycan biosynthesis	POMT1
21. hsa-miR-151a-3p		Hicks et al., 2020; Mundalil Vasu et al., 2014	Glycosaminoglycan degradation	GNS, IDS
22. hsa-miR-155-5p	Almehmadi et al., 2020; Wu et al., 2016; Mor et al., 2015;		Other glycan degradation	NEU1, GLB1
23. hsa-miR-338	Mor et al., 2015; Ghahramani Seno et al., 2011	Schumann et al., 2017	Glycosaminoglycan biosynthesis - keratan sulfate	B3GNT1, B3GNT2
24. has-miR-3687		Kichukova et al., 2017; Wu et al., 2016	Amino sugar and nucleotide sugar metabolism	HK1
25. miR-423-5p	Jyonouchi et al., 2019	Bleazard, 2018; Kim et al., 2023	N-Glycan biosynthesis<br><br>Glycosaminoglycan biosynthesis - keratan sulfate	RPN1, DPM2, B4GALT1, ALG10B, ALG14, MAN1B1, DDOST, DOLK, B4GALT3, MGAT1, ALG3, MGAT4B<br><br>Keratan: ST3GAL1, B4GALT1, B4GALT3

Supplementary Table S2: 9 miRNAs reported to be significantly differentially expressed (DE) in a ASD cellular context.

miRNA	Upregulated (References)	Downregulated (References)	Glycosylation Pathways	Targeted Glycogenes
hsa-miR-21-3p	Mor et. al 2015	Abu-Elneel et al., 2008	Glycosaminoglycan biosynthesis - heparan sulfate / heparin	EXT1, EXTL3, NDST2
hsa-miR-199a-5p	Vaccaro et al., 2018; Ghahramani Seno et al., 2011		Glycosaminoglycan biosynthesis - heparan sulfate / heparin	EXT1
hsa-miR-195-5p	Vasu et al., 2014; Sarachana et al., 2010	Huang et al., 2015	Glycosaminoglycan biosynthesis - chondroitin sulfate / dermatan sulfate<br><br>Mucin type O-Glycan biosynthesis	UST, DSE, CHPF, CHPF2<br><br>GALNT7, GALNT1, GALNT3, GALNT2
hsa-miR-132-3p	Talebizadeh et al., 2008	Sarachana et al., 2010; Abu-Elneel et al., 2008	Other types of O-glycan biosynthesis	LFNG, B3GAT1, POMT1, EOGT

miR-132-5p	Talebizadeh et al., 2008; Kim et al., 2023	Sarachana et al., 2010; Abu-Elneel et al., 2008	Glycosaminoglycan biosynthesis - keratan sulfate<br><br>N-Glycan biosynthesis<br><br>Other types of O-glycan biosynthesis	B4GALT1, B3GNT1<br><br>GANAB, B4GALT1<br><br>B4GALT1
hsa-miR-30c-5p	Sarachana et al., 2010	Hicks et al., 2016	Mucin type O-Glycan biosynthesis	GALNT7, B4GALT5, ST3GAL1, GCNT3,<br><br>GALNT1, GALNT3, GALNT2
hsa-miR-379-5p	Mor et al., 2015; Hicks et al., 2020		Mucin type O-Glycan biosynthesis	GALNT11
has-miR-1277-3p	Wu et al. 2020; Winden et al., 2018		Glycosaminoglycan biosynthesis - keratan sulfate<br><br>Glycosaminoglycan biosynthesis - heparan sulfate	CHST1, B3GNT2<br><br>EXT1
miR-423-5p		Bleazard, 2018;<br><br>Kim et al., 2023	N-Glycan biosynthesis<br><br>Glycosaminoglycan biosynthesis - keratan sulfate	RPN1, DPM2, B4GALT1, ALG10B, ALG14, MAN1B1, DDOST, DOLK, B4GALT3, MGAT1,

				ALG3, MGAT4B<br><br>Keratan: ST3GAL1, B4GALT1, B4GALT3

Supplementary Table S3: Degree and Betweenness values from the whole miRNA-mediated regulatory network revealed by miRNet tool.

For each gene-node of the network reconstructed using the 9 miRNAs identified in study as input, we reported the values of degree, which represent the number of connections the node has to other nodes, and the values of betweenness, which measure the number of the shortest paths going through the node. Nodes with higher degree act as important "hubs", and with a higher betweenness act as important "bottlenecks" in a network. Among the most relevant gene-nodes, we identified the glycogene *B4GALT1*, with high degree and betweenness values.

ID	Degree	Betweenness
hsa-mir-195-5p	1498	1173694
hsa-mir-30c-5p	1152	728381.6
hsa-mir-21-3p	970	530614.4
hsa-mir-423-5p	737	349514.5
hsa-mir-132-3p	690	292651.8
hsa-mir-199a-5p	315	82378.27
hsa-mir-379-5p	111	14951.8
hsa-mir-132-5p	94	10462.18
hsa-mir-1277-3p	54	5463.06
NFIB	7	1905.427
HNRNPA2B1	6	1323.719
SLC7A5	6	1551.977
TNRC6A	6	1727.231
PCGF5	6	1727.231
DDX21	6	1440.112
HIPK2	6	1551.977
NRP1	6	1551.977
CD44	5	856.8098
CLTC	5	1127.37
HSPA5	5	941.639
B4GALT1	5	1056.357
HNRNPU	5	1126.65
POU2F1	5	941.639
CSDE1	5	1126.65
CELF1	5	1298.183
LARP1	5	1198.732
IPO9	5	1298.183
CPEB4	5	1298.183
SMAD2	5	1298.183
APP	5	1127.37

Supplementary Table S4: Degree and Betweenness values from a miRNA-mediated regulatory network revealed by miRNet tool and reconstructed from a subset of miRNAs that impact N-glycosylation pathway.

In this supplementary network analysis, we considered ASD-miRNAs that are simultaneously involved in N-glycosylation pathway (i.e. miR-106b-5p, miR-423-5p and miR-132-5p).

For each gene-node of the network reconstructed, we reported here the values of degree, which represent the number of connections the node has to other nodes, and the values of betweenness, which measure the number of the shortest paths going through the node. Nodes with higher degree act as important "hubs", and with a higher betweenness act as important "bottlenecks" in a network. Out of the most relevant gene-nodes, glycogene *B4GALT1* exhibits good degree and betweenness values.

**ID**	**Degree**	**Betweenness**
hsa-mir-106b-5p	372	38439.81
hsa-mir-423-5p	372	38439.81
hsa-mir-132-5p	66	1310.379
ADAR	3	7.935234
NID1	3	7.935234
SOD2	3	7.935234
BTG2	3	7.935234
ULK1	3	7.935234
NCAPD2	3	7.935234
BCL2L11	3	7.935234
SPEN	3	7.935234
TRIM41	3	7.935234
PRR14L	3	7.935234
RGMB	3	7.935234
NFE2L2	3	7.935234
SLC7A5	3	7.935234
DDX21	3	7.935234
HNRNPA2B1	3	7.935234
SRSF6	3	7.935234
PHF10	3	7.935234
PREPL	3	7.935234
TSC22D3	3	7.935234
MSL1	3	7.935234
B4GALT1	2	3.907511

Supplementary Table S5: Degree and Betweenness values from a miRNA-mediated regulatory network revealed by miRNet tool and reconstructed from a subset of miRNAs that impact Mucin type O-glycosylation pathway.

In this supplementary network analysis, we considered ASD-miRNAs that are simultaneously involved in Mucin type O-glycosylation pathway (miR-27a-3p, miR-30c-5p, miR-195-5p, miR-30e-5p and miR-379-5p). For each gene-node of the network reconstructed, we reported here the values of degree, which represent the number of connections the node has to other nodes, and the values of betweenness, which measure the number of the shortest paths going through the node. Nodes with higher degree act as important "hubs", and with a higher betweenness act as important "bottlenecks" in a network. Among the most relevant gene-nodes, we identified the glycogenes *GALNT3* and *GALNT2*, with good degree and betweenness values.

ID	Degree	Betweenness
hsa-mir-27a-3p	1856	1401563
hsa-mir-30c-5p	1738	1094236
hsa-mir-195-5p	1597	996177.4
hsa-mir-30e-5p	1552	823146.4
hsa-mir-379-5p	108	6673.14
TAOK1	5	858.1777
C5orf51	5	858.1777
MCL1	5	858.1777
AGO2	5	858.1777
NFIB	5	858.1777
SLC20A1	5	858.1777
MICAL2	5	858.1777
RBM12B	5	858.1777
CCNA2	5	858.1777
PRPF40A	5	858.1777
GALNT3	4	742.0274
GALNT2	4	742.0274

Supplementary Table S6: Degree and Betweenness values from a miRNA-mediated regulatory network revealed by miRNet tool and reconstructed from a subset of miRNAs that impact other types of O-glycosylation.

In this supplementary network analysis, we considered ASD-miRNAs that are simultaneously involved in other types of O-glycolylation. For each gene-node of the network reconstructed, we reported here the values of degree, which represent the number of connections the node has to other nodes, and the values of betweenness, which measure the number of the shortest paths going through the node. Nodes with higher degree act as important "hubs", and with a higher betweenness act as important "bottlenecks" in a network. Among the most relevant gene-nodes, the glycogenes *POMT1, B4GALT1, OGT* and *LFNG* show good degree and betweenness values.

ID	Degree	Betweenness
hsa-let-7a-5p	546	106215.5
hsa-mir-132-3p	442	59459.44
hsa-mir-144-3p	213	14963.01
hsa-mir-132-5p	57	1257.088
BCL2L11	4	46.82757
BTG2	4	46.82757
RGMB	4	46.82757
POMT1	3	5.272787
B4GALT1	2	9.423383
OGT	2	0.5931569
LFNG	2	0.5931569

Supplementary Table S7: Degree and Betweenness values from a miRNA-mediated regulatory network revealed by miRNet tool and reconstructed from a subset of miRNAs that impact Heparan Sulphate biosynthesis pathway.

ASD-miRNAs that are simultaneously involved in Heparan Sulphate biosynthesis pathway (mir-21-3p, mir-199a-5p and mir-1277-3p). For each gene-node of the network reconstructed, we reported here the values of degree, which represent the number of connections the node has to other nodes, and the values of betweenness, which measure the number of the shortest paths going through the node. Nodes with higher degree act as important "hubs", and with a higher betweenness act as important "bottlenecks" in a network. The glycogene *EXT1* exhibits a good degree and betweenness values.

ID	Degree	Betweenness
hsa-mir-21-3p	110	3832.133
hsa-mir-199a-5p	97	2487.755
hsa-mir-1277-3p	20	120.1125
EXT1	3	9.557189
EXTL3	2	0.2009306

References

1. Abdelkarem, O. A. I., Zaki, M. A., Elwafa, R. A. H. A., Elmaksoud, M. A., & El Banna, A. (2024). Evaluation of the diagnostic performance of circulating microRNAs for the diagnosis of autism spectrum disorders. Alexandria journal of pediatrics, 37(2), 130–136. https://doi.org/10.4103/ajop.ajop_21_24
2. Abu-Elneel, K., Liu, T., Gazzaniga, F. S., Nishimura, Y., Wall, D. P., Geschwind, D. H. et al. (2008). Heterogeneous dysregulation of microRNAs across the autism spectrum. Neurogenetics, 9(3), 153–161. https://doi.org/10.1007/s10048-008-0133-5
3. Almehmadi, K. A., Tsilioni, I., & Theoharides, T. C. (2020). Increased expression of miR-155p5 in amygdala of children with autism spectrum disorder. Autism research : official journal of the International Society for Autism Research, 13(1), 18–23. https://doi.org/10.1002/aur.2205
4. Bleazard, Thomas. "Investigating the role of microRNAs in autism." (2018). Corpus ID: 149752101
5. Ghahramani Seno, M. M., Hu, P., Gwadry, F. G., et al. (2011). Gene and miRNA expression profiles in autism spectrum disorders. Brain Research, 1380, 85–97. https://doi.org/10.1016/j.brainres.2010.09.046
6. Hicks, S. D., & Middleton, F. A. (2016). A comparative review of microRNA expression patterns in autism spectrum disorder. Frontiers in Psychiatry, 7, 176. https://doi.org/10.3389/fpsyt.2016.00176
7. Hicks, S. D., Carpenter, R. L., Wagner, K. E., Pauley, R., Barros, M., Tierney-Aves, C. et al. (2020). Saliva microRNA differentiates children with autism from peers with typical and atypical development. Journal of the American Academy of Child & Adolescent Psychiatry, 59(2), 296–308. https://doi.org/10.1016/j.jaac.2019.03.017
8. Huang, F., Long, Z., Chen, Z., Li, J., Hu, Z., Qiu, R. et al. (2015). Investigation of gene regulatory networks associated with autism spectrum disorder based on miRNA expression in China. PLoS one, 10(6), e0129052. https://doi.org/10.1371/journal.pone.0129052
9. Huang, Z. X., Chen, Y., Guo, H. R., & Chen, G. F. (2021). Systematic review and bioinformatic analysis of microRNA expression in autism spectrum disorder identifies pathways associated with cancer, metabolism, cell signaling, and cell adhesion. Frontiers in Psychiatry, 12, 630876. https://doi.org/10.3389/fpsyt.2021.630876
10. Jyonouchi, H., & Geng, L. (2019). Associations between monocyte and T cell cytokine profiles in autism spectrum disorders: Effects of dysregulated innate immune responses on adaptive responses to recall antigens in a subset of ASD children. International journal of molecular sciences, 20(19), 4731. https://doi.org/10.3390/ijms20194731
11. Jyonouchi, H., Geng, L., Streck, D. L., Dermody, J. J., & Toruner, G. A. (2017). MicroRNA expression changes in association with changes in interleukin-1ß/interleukin10 ratios produced by monocytes in

autism spectrum disorders: Their association with neuropsychiatric symptoms and comorbid conditions (observational study). Journal of neuroinflammation, 14, 229.

12. Kichukova, T. M., Popov, N. T., Ivanov, I. S., & Vachev, T. I. (2017). Profiling of circulating serum microRNAs in children with autism spectrum disorder using stem-loop qRT-PCR assay. Folia medica, 59(1), 43–52. https://doi.org/10.1515/folmed-2017-0009
13. Kim, J. Y., Kim, W., & Lee, K. H. (2023). The role of microRNAs in the molecular link between circadian rhythm and autism spectrum disorder. Animal cells and systems, 27(1), 38–52. https://doi.org/10.1080/19768354.2023.2180535
14. Mor, E., & Shomron, N. (2013). Species-specific microRNA regulation influences phenotypic variability: perspectives on species-specific microRNA regulation. BioEssays : news and reviews in molecular, cellular and developmental biology, 35(10), 881–888. https://doi.org/10.1002/bies.201200157
15. Mor, M., Nardone, S., Sams, D. S., & Elliott, E. (2015). Hypomethylation of miR-142 promoter and upregulation of microRNAs that target the oxytocin receptor gene in the autism prefrontal cortex. Molecular autism, 6, Article 16. https://doi.org/10.1186/s13229-015-0012-5
16. Mundalil Vasu, M., Anitha, A., Thanseem, I., Suzuki, K., Yamada, K., Takahashi, T. et al. (2014). Serum microRNA profiles in children with autism. Molecular autism, 5, 40. https://doi.org/10.1186/2040-2392-5-40
17. Nakata, M., Kimura, R., Funabiki, Y., Awaya, T., Murai, T., & Hagiwara, M. (2019). MicroRNA profiling in adults with high-functioning autism spectrum disorder. Molecular brain, 12. https://doi.org/10.1186/s13041-019-0508-6Safdar, A.; Khurshid, S.; Farwa, U.; Bakhtiar, S. M. The Analyzing MiR-106b-5p and MiR-93-5p As Promising Diagnostic Markers for Autism Spectrum Disorder. *CTO* **2021**, *1*, 36-44.
18. Salloum-Asfar, S., Elsayed, A. K., Elhag, S. F., & Abdulla, S. A. (2021). Circulating non-coding RNAs as a signature of autism spectrum disorder symptomatology. International journal of molecular sciences, 22(12), 6549. https://doi.org/10.3390/ijms22126549
19. Sarachana T, Zhou R, Chen G, Manji HK, Hu VW. Investigation of post-transcriptional gene regulatory networks associated with autism spectrum disorders by microRNA expression profiling of lymphoblastoid cell lines. Genome Med. 2010;2(4):23. Published 2010 Apr 7. doi:10.1186/gm144
20. Schumann, C. M., Sharp, F. R., Ander, B. P., & Stamova, B. (2017). Possible sexually dimorphic role of miRNA and other sncRNA in ASD brain. Molecular autism, 8, 4. https://doi.org/10.1186/s13229-017-0117-0
21. Talebizadeh, Z., Butler, M. G., & Theodoro, M. F. (2008). Feasibility and relevance of examining lymphoblastoid cell lines to study role of microRNAs in autism. Autism Research, : official journal of the International Society for Autism Research 1(4), 240–250. https://doi.org/10.1002/aur.33

22. Vaccaro, T. D. S., Sorrentino, J. M., Salvador, S., Veit, T., Souza, D. O., & De Almeida, R. F. (2018). Alterations in the MicroRNA of the blood of autism spectrum disorder patients: Effects on epigenetic regulation and potential biomarkers. Behavioral sciences, 8(8), 75.
23. Winden, K. D., Ebrahimi-Fakhari, D., & Sahin, M. (2018). Abnormal mTOR Activation in Autism. Annual review of neuroscience, 41, 1–23. https://doi.org/10.1146/annurev-neuro-080317-061747
24. Wu, Y., Parikshak, N., Belgard, T. & Geschwind, D. H. (2016). Genome-wide, integrative analysis implicates microRNA dysregulation in autism spectrum disorder. Nature neuroscience, 19, 1463–1476. https://doi.org/10.1038/nn.4342
25. Wu, X., Li, W., & Zheng, Y. (2020). Recent Progress on Relevant microRNAs in Autism Spectrum Disorders. International Journal of Molecular Sciences, 21(16), 5904. https://doi.org/10.3390/ijms21165904
